# Ovarian PERK/NRF2/CX43/StAR/progesterone pathway activation mediates female reproductive dysfunction induced by cold exposure

**DOI:** 10.1038/s41598-024-60907-9

**Published:** 2024-05-04

**Authors:** Mengnan Ding, Yarong Lu, Qing Wen, Chen Xing, Xin Huang, Yifan Zhang, Wei Wang, Chongchong Zhang, Min Zhang, Fanfei Meng, Kun Liu, Guangchao Liu, Lun Song

**Affiliations:** 1 Beijing Institute of Basic Medical Sciences, Beijing, 100850 China; 2 College of Life Science, Henan Normal University, 46 Jianshe Road, Xinxiang, 473007 China; 3 School of Pharmacy, Jiamusi University, Jiamusi, 154007 China; 4 Anhui Medical University, 81 Meishan Road, Hefei, 230032 China; 5 Henan University Joint National Laboratory for Antibody Drug Engineering, Henan, 465004 China

**Keywords:** Cold, PERK, NRF2, CX43, Progesterone, Female reproductive disorders, Endocrine reproductive disorders, Risk factors

## Abstract

Ambient air temperature is a key factor affecting human health. Female reproductive disorders are representative health risk events under low temperature. However, the mechanism involving in cold-induced female reproductive disorders remains largely unknown. Female mice were intermittently exposed to cold conditions (4 °C) to address the health risk of low temperature on female reproductive system. Primary granulosa cells (GCs) were prepared and cultured under low temperature (35 °C) or exposed to β3-adrenoreceptor agonist, isoproterenol, to mimic the condition of cold exposure. Western-blot, RT-PCR, co-IP, ELISA, pharmacological inhibition or siRNA-mediated knockdown of target gene were performed to investigate the possible role of hormones, gap conjunction proteins, and ER stress sensor protein in regulating female reproductive disorders under cold exposure. Cold exposure induced estrous cycle disorder and follicular dysplasia in female mice, accompanying with abnormal upregulation of progesterone and its synthetic rate-limiting enzyme, StAR, in the ovarian granulosa cells. Under the same conditions, an increase in connexin 43 (CX43) expressions in the GCs was also observed, which contributed to elevated progesterone levels in the ovary. Moreover, ER stress sensor protein, PERK, was activated in the ovarian GCs after cold exposure, leading to the upregulation of downstream NRF2-dependent *CX43* transcription and aberrant increase in progesterone synthesis. Most importantly, blocking PERK expression in vivo significantly inhibited NRF2/CX43/StAR/progesterone pathway activation in the ovary and efficiently rescued the prolongation of estrous cycle and the increase in follicular atresia of the female mice induced by cold stress. We have elucidated the mechanism of ovarian PERK/NRF2/CX43/StAR/progesterone pathway activation in mediating female reproductive disorder under cold exposure. Targeting PERK might be helpful for maintaining female reproductive health under cold conditions.

## Introduction

With the increasing number of women working or travelling in the cold environment (such as high altitude, polar region), the threat on the women’s reproductive health posed by cold conditions is considered as a big challenge^1–5^. Multiple experimental evidences have revealed that short or long exposing to low temperature usually lead to significant reproductive dysfunction in female mice or rats, including sympathetic nervous system activation-induced irregular estrous cycle^1,3,5^, abnormal hormone secretion or ovarian hormone receptors expression^2,3,5^, ovarian morphological changes^3^, follicular dysplasia^1,5^, ovarian and uterine microcirculation disorders^1^, and the phenotype of polycystic ovarian syndrome (PCOS)^4,5^. However, the mechanisms involving in cold-induced female reproductive disorders remain largely unknown.

Female reproductive function is tightly controlled by the coordinated activities of the hypothalamus–pituitary–ovary (HPO) axis. The sympathetic signals and gonadotropins released by the hypothalamus regulate the synthesis and release of the follicle-stimulating hormone (FSH) and luteinizing hormone (LH) in the pituitary gland. FSH and LH then stimulate the reproductive events occurred in the ovary, uterus and oviduct, including follicular growth and differentiation, steroid hormone synthesis and secretion, ovulation, implantation, and parturition^6,7^. As one of the key components of female reproductive system, ovary has the dual functions of releasing mature oocytes and synthesizing endocrine hormones (such as estrogen, progesterone). Follicles are the functional units of the ovary, which are composed of three key cell populations: an oocyte, surrounded by supporting granulosa cells (GCs), themselves surrounded by theca cells. The coordinated activity of oocytes, granulosa cells and theca cells is essential to maintaining follicular development and ovarian homeostasis^8,9^. Any deregulation of HPO axis and the correlated signal events will result in menstrual cycle disorder, abnormal follicular development, impaired ovulation and even decreased fertility^7^.

Gap junctions are membrane channels found in all cells of the human body that are essential to cellular physiology. Gap junctions are formed from connexin proteins and are responsible for transfer of biologically active molecules, metabolites, and salts between neighboring cells or cells and their extracellular environment. In mammalian ovaries, gap junctions formed by connexin proteins can couple the granulosa cells with each other and with the oocyte, establishing a metabolic syncytium crucial to oogenesis. Studies on mice and various other species have demonstrated that connexin 43 (CX43) is the predominant CX in the gap junctions formed between ovarian granulosa cells to mediate the connection and communication between adjacent cells. CX37 localizes to gap junctions between oocyte and granulosa cells, which are responsible for transfer of amino acids, glucose, ions and cGMP from the granulosa cells to oocyte to support the growing and metabolism of oocyte and maintain the oocyte in a state of meiotic arrest. The decreased expression of CX during the ovulation attenuates the communication between the oocyte and granulosa cells and reduces cGMP level in the oocyte, thus allowing it to complete meiosis. Therefore, CX protein levels play a pivotal role in follicular development and oocyte growth^9,10^.

Besides connexin, endoplasmic reticulum (ER) stress sensor proteins are also considered to be the key regulators of follicular development and ovarian function^11,12^. ER stress is commonly induced in the granulosa cells by a variety of physiological and pathological conditions, and then triggers GCs apoptosis^13–16^. The protein kinase RNA-like endoplasmic reticulum kinase (PERK) is one of the ER stress sensor proteins that is attracted most attention in the regulating of ovarian physiology. Activation of PERK can lead to phosphorylation of α subunit of eukaryotic initiation factor 2 (eIF2α) and the transcription of activating transcription factor 4 (ATF4) which regulates the expression of target genes such as the CCAAT/enhancer-binding protein homologous protein (CHOP). The PERK/eIF2α/ATF4/CHOP signaling pathway is considered to play a pivotal role in GCs apoptosis associated with ER stress activation in the ovary^13–17^. However, whether PERK is involved in cold-induced ovarian disorder is still unknown.

In the current study, we investigated the molecular mechanism involving in mediating female reproductive disorders upon low temperature by focusing on the role of PERK, CX43 and the endocrine hormones.

## Methods

### Animals experiments

Female c57BL/6 mice aged at 8 weeks were purchased from Charles River Laboratory Animal Technology Co (Beijing, China). Mice were bred in animal facilities under specific pathogen-free (SPF) conditions for a week, and were provided with standard rodent chow and water every day. Mice were housed in a temperature-controlled room at 23 ± 1 °C under a 12–12 h light–dark cycle with food and water ad libitum. Morning 07:00 (zeitgeber time) was considered as the ZT0. Cold exposure was performed at 4 °C, 4 h per day (10:00 to 14:00; n = 20) for 3 days. After 3 days, all mice were euthanized at ZT0 by cervical dislocation. At least three independent experiments were conducted in this study. Mice studies were strictly complied with the ARRIVE guidelines and were carried out in accordance with the National Research Council's Guide for the Care and Use of Laboratory Animals. The protocols of all animal experiments were approved by the Ethics Committee of Beijing Institute of Basic Medical Sciences.

### Reagents

Recombinant follicle stimulating hormone (FSH) (PRS-HOR-253) was supplied by ProSpec Ltd. (Ness-Ziona, Israel), PERK Inhibitor GSK2606414 (1337531-89-1) were purchased from Millipore (USA). Transfections were performed with the Lipofectamine TM RNAi MAX (Invitrogen, 13778-150) and Opti-MEM medium (Gibco, 11058021) according to the manufacturer’s instructions. The siRNAs specific targeting PERK, NRF2, CX43 and their control siRNAs were designed and synthesized by RiboBio Biotechnology (Guangzhou, China). Hyaluronidase (37326-33-3) was purchased from Solarbio (Beijing, China).

### Estrous cycle determination

Mice estrous cycle was monitored via daily vaginal smears observed under a light microscope according to our previous published protocol^18^.

### Ovarian follicles histological analysis

The ovaries of control and cold-treated mice were collected and subjected to HE staining. The section in the center of the ovary of each mouse were selected to guarantee the sections comparable in the control and cold-treated mice. Then, follicles categorization and the percentage of the atretic follicles in the ovaries were examined as described previously^18^.

### Granulosa cells (GCs) culture, transfection and treatment

Primary GCs were prepared as described previously^18^. To knockdown the expression of the target genes, specific siRNA or its control siRNA were transfected into GCs using LipofectAMINE™RNAi MAX according to the manufacturer’s instructions. For cold stress experiments, GCs were cultured under mild cold conditions (35 °C) for 12 h, taking the cells cultured under normal temperature (37 °C) as the control. Or GCs were treated with β3-adrenoreceptor agonist, isoproterenol (10 μM), for 4 h to mimic the condition of sympathetic activation under cold exposure, taking the cells treated with DMSO as the control. Cell lysate and cell culture supernatants were collected for the next step detection.

### Serum PG assay

Progesterone (PG) levels in the mice blood samples or GCs culture supernatants were measured using the ELISA kits (CEA459Ge, CLOUD-CLONE CORP, China) according to the protocol provided by the manufacturer. Because the level of progesterone shows circadian rhythmicity and change significantly during the estrous cycle, we measured the level of progesterone at 7 am during estrus for both control and cold exposure mice in each experiment.

### RT-PCR assay

Total RNA was extracted with TRIzol reagent (Sigma-Aldrich, T9424) and the cDNA were synthesized with the Thermo Script-TM RT-PCR system (Thermo Fisher Scientific, M1631). To analyze the induction of *CX43* transcription, the following oligonucleotides were synthesized and used as specific primers to amplify the target cDNAs: 5′-cacgcccagccgtttgattt3′ (forward) and 5′-ctctgctggaaggtcgctga-3′ (reverse); The primers used to amplify *GAPDH* cDNA were 5′-agctatgcgctgcctgacgg-3′ (forward) and 5′-gcagctcagtaacagtccgc-3′ (reverse).

### Western blot assay

Western blot assays were performed as described previously^18^. Due to the diversity of the target proteins to be tested, the membrane was usually cropped into 2–4 parts based on the molecular weight of the target proteins and then the corresponding antibody hybridization was performed. The primary antibodies used in this study included p-PERK (1:1000, Abclonal, AP0886), PERK (1:1000, Cell Signaling Technology, 2683), p-NRF2 (1:1000, Affinity, DF7519), NRF2 (1:1000, Cell Signaling Technology, 12721), p-STAT3 (1:1000, Cell Signaling Technology, 9139), STAT3 (1:1000, Cell Signaling Technology, 9145), p-c-Jun (1:1000, Cell Signaling Technology, 3270), c-Jun (1:1000, Cell Signaling Technology, 9165), MyoD (1:200, Santa Cruz Biotechnology, sc-377460), connexin 43 (1:2000, Proteintech, 26980-1-AP), CYP11A1 (1:1000, Cell Signaling Technology, 14217), CYP19A1 (1:1000, Abcam, ab18995), StAR (1:1000, Cell Signaling Technology, 8449s), and β-actin (1:5000, Proteintech, 20536-1-AP). The data were obtained from triplicates of each independent experiment. Raw data for each blot were shown in the Supplementary file.

### Statistics

Data were tested for significance employing the sample *t* test, and presented as mean ± SD. Data was analyzed by SPSS 19.0 and Graphpad Prime 6 software, P < 0.05 indicated that the difference was statistically significant.

### Ethics approval and consent to participate

The protocols of all animal experiments were approved by the Ethics Committee of Beijing Institute of Basic Medical Sciences (Document No. IACUC-DWZX-2021-533).

## Results

### Cold exposure induced estrous cycle disorder and follicular dysplasia in female mice

**Figure 1:**
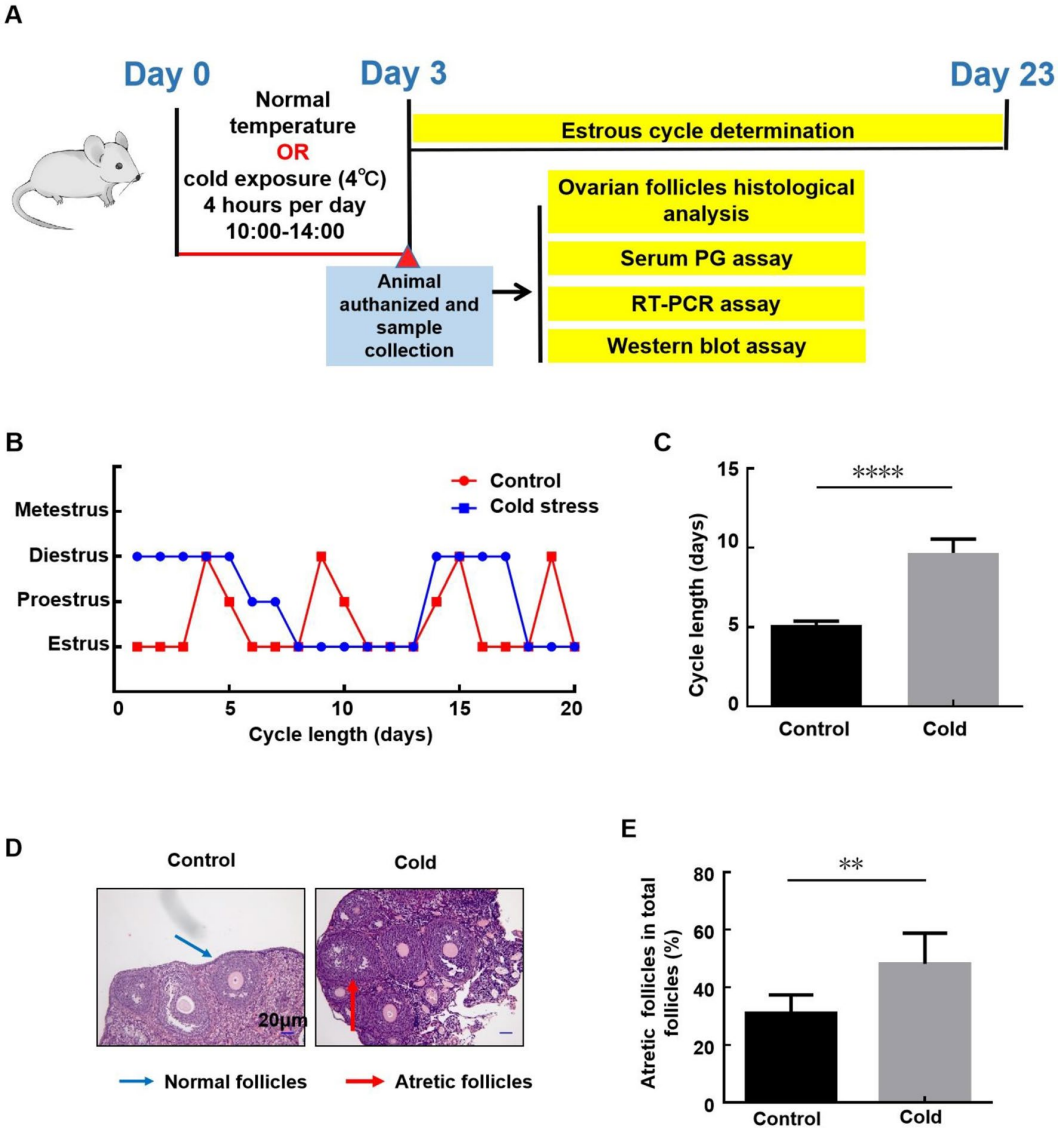
Cold exposure induced estrous cycle disorder and follicular dysplasia in female mice. (**A**) The flow diagram of the animal experimental design. (**B**,**C**) 8-week-old unmated female mice were left untreated or exposed to cold conditions (4 °C, ZT3-ZT7, 4 h/day, 3 consecutive days), and then the estrous cycle of mice in each group was examined in the following 3 weeks. The distribution of estrous cycles of representative untreated and cold stress-treated mice was shown in (**B**). And the average cycle length of mice from each group was shown in (**C**) (n = 18, ****p < 0.0001). (**D**,**E**) The mice were euthanized after 3 days of intermittent cold exposure, and the ovaries were collected and subjected to HE staining. The follicles categorization was performed and the percentage of atretic follicles in the total follicles was calculated (n = 6, **p < 0.01).

To address the health risk of low temperature on female reproductive system, 8-week-old unmated female mice were normally raised or intermittently exposed to cold conditions (4 °C, ZT3-ZT7, 4 h/day, 3 consecutive days), and then the index of female reproductive functions were examined (Fig. 1A). Here we found that cold exposure significantly prolonged the estrous cycle of female mice (Fig. 1B). The cycle length increased from about 5 days under normal condition to about 9.5 days after cold exposure (Fig. 1C). Under the same conditions, a significant increase in the percentage of the atretic follicles in the ovaries of the cold-stressed mice was observed (Fig. 1D,E). These data indicate that cold exposure induces estrous cycle disorder and follicular dysplasia in female mice.

### Cold exposure induced StAR-dependent progesterone upregulation in female mice

**Figure 2:**
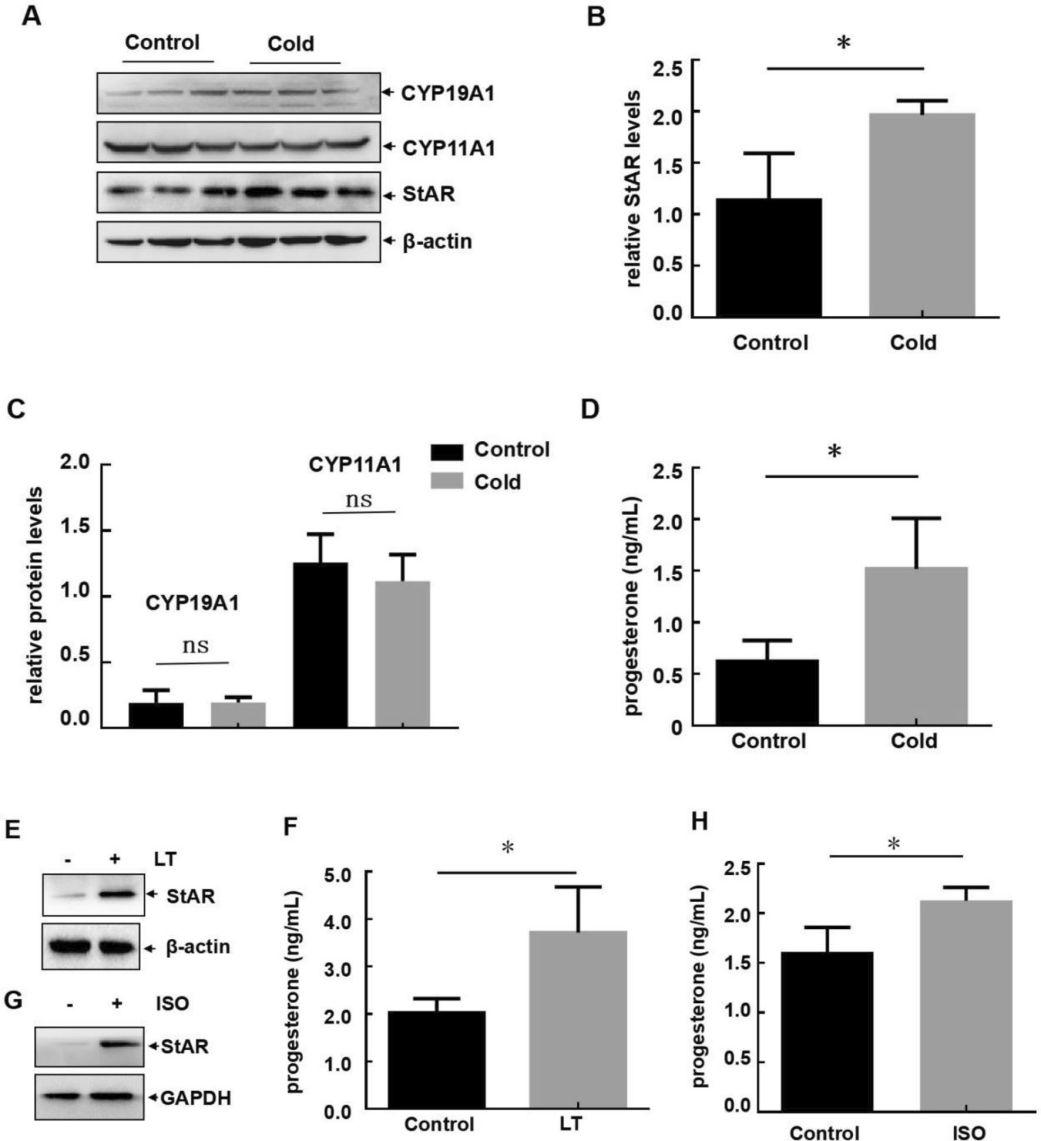
Cold exposure induced StAR-dependent progesterone upregulation in female mice. (**A**–**C**) Female mice were untreated or exposed to cold stress as described in Fig. 1A. The ovaries of the mice in each group were collected and subjected to western-blot assay to detect the expression levels of the rate limiting enzymes for progesterone synthesis (StAR) and estrogen synthesis (CYP11A1 and CYP19A1). The quantitative results of each target protein (relative to β-actin) were also shown in (**B**,**C**). (**D**) The serum levels of progesterone at 7 am during estrus in the control and cold stress-treated mice were analyzed by ELISA. (**E**,**F**) The primary granulosa cells (GCs) were extracted from the ovaries and then cultured at normal (37 °C) or low temperature (LT, 35 °C). Then StAR expression (**E**) and progesterone levels in the cell culture supernatants (**F**) were detected. (**G**,**H**) The primary GCs were treated with isoproterenol (ISO) and then StAR expression (**G**) and progesterone levels in the cell culture supernatant (**H**) were detected (n = 6, *p < 0.05).

To disclosing the mechanism involving in cold-induced estrous cycle disorder and follicular dysplasia in female mice, we next examined whether the potential responses leading to female reproductive dysfunction existed in the cold-treated female mice, such as disorder of estrogen or progesterone expression by granulosa cells, abnormal changes on gonadotropin receptors or gap conjunction proteins expression in the follicles, etc.^6,8,9^. We observed that the levels of the rate limiting enzymes for progesterone synthesis, StAR, increased in the ovary of the mice upon cold exposure (Fig. 2A,B), which was consistent with the upregulation of serum progesterone levels under the same conditions (Fig. 2D). However, the levels of the enzymes responsible for estrogen synthesis, CYP11A1 and CYP19A1, remained unchanged after cold exposure (Fig. 2A,C). These data indicated that cold exposure induced StAR-dependent progesterone upregulation in female mice.

To further confirm the above results, the primary granulosa cells (GCs), by which progesterone is synthesized, were extracted from the ovaries and then cultured at normal (37 °C) or low temperature (LT, 35 °C). As shown in Fig. 2E,F, StAR expression and progesterone synthesis were dramatically upregulated in the primary granulosa cells exposed to 35 °C compared to culture at 37 °C. Moreover, when the GCs were treated with the β3-adrenoreceptor agonist, isoproterenol (ISO), to mimic the condition of sympathetic activation under cold exposure, we also observed the increase in StAR expression and progesterone synthesis (Fig. 2G,H). These data suggested that the upregulation of StAR-dependent progesterone in ovarian granulosa cells might play a crucial role in cold-induced female reproductive disorders.

### Cold exposure-induced connexin 43 upregulation led to StAR-dependent progesterone elevation in the ovarian granulosa cells

**Figure 3:**
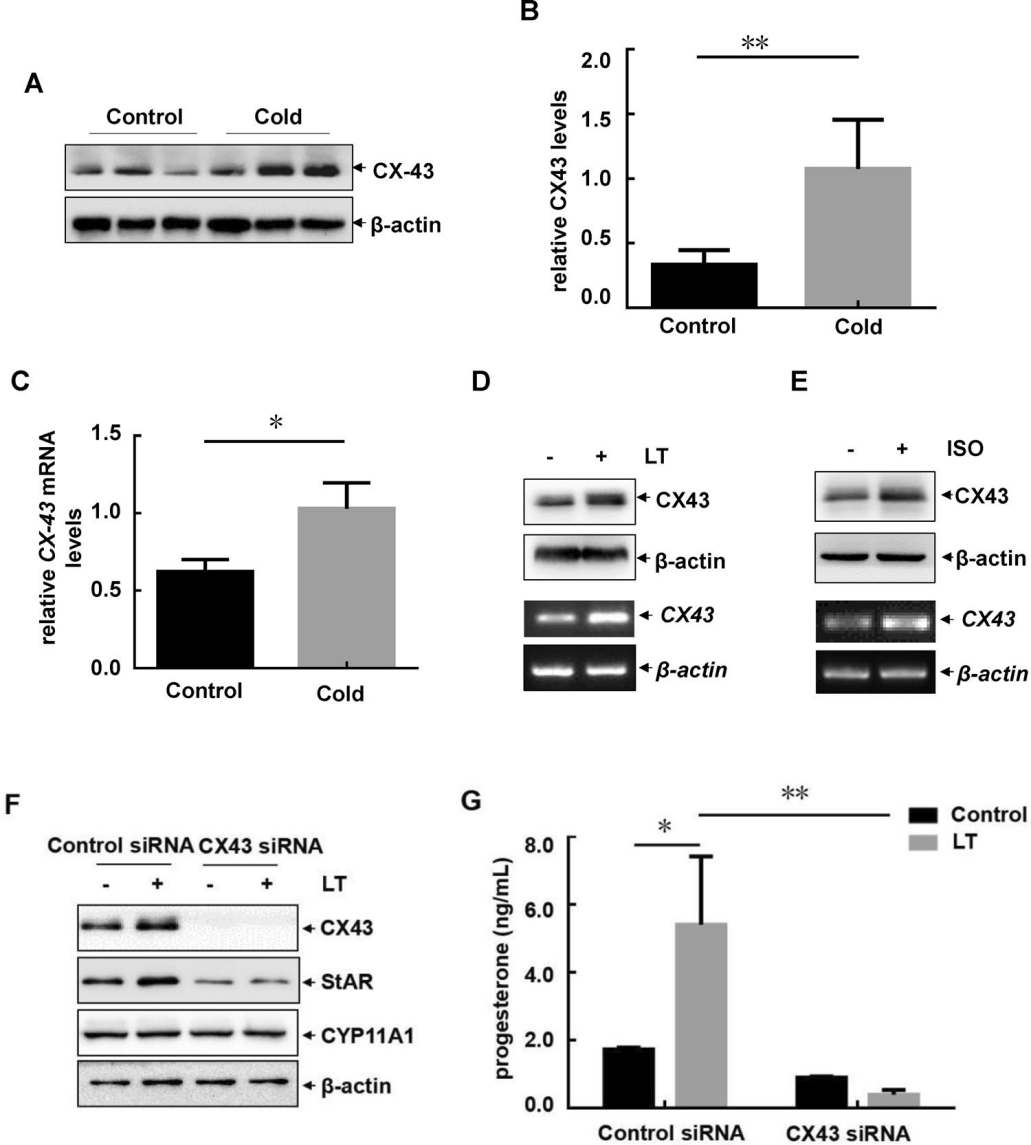
Cold exposure-induced connexin 43 upregulation led to StAR-dependent progesterone elevation in the ovarian granulosa cells. (**A**–**C**) Female mice were untreated or exposed to cold stress as described in Fig. 1A. The ovaries of the mice in each group were collected and subjected to western-blot (**A**) or real-time PCR (**C**) assay to detect the expression levels of CX43. The quantitative results of CX43 levels relative to β-actin were also shown in (**B**). (**D**,**E**) The primary granulosa cells (GCs) were extracted and treated as described in Fig. 2E,G. Then the protein and RNA levels of CX43 were detected. (**F**,**G**) The primary GCs were transfected with CX43 siRNA or their control siRNAs, followed by exposure to cold stress for 12 h. Then the changes on StAR expression (**F**) and the progesterone levels in cell culture supernatants (**G**) were detected (**p < 0.01, *p < 0.05).

Gap conjunction proteins expressing on the oocyte, granulosa cells and zonal pellucida are key regular of the female reproductive function^9,10^. Connexin 43 (CX43), one of the gap conjunction proteins mainly expresses on the GCs, play a critical role in maintaining ovulation and regular estrous cycle^10^. Here we found that cold exposure induced an increase of CX43 expression in the ovary (Fig. 3A,B), accompanying with the upregulation of *CX43* transcription under the same conditions (Fig. 3C). In addition, the enhancement of CX43 transcription and protein synthesis was also observed in the primary GCs exposed to low temperature or isoproterenol (Fig. 3D,E). Most importantly, knockdown of CX43 expression blocked StAR upregulation in the GCs induced by cold exposure (Fig. 3F). Under the same conditions, the elevation of progesterone synthesis and secretion was also significantly inhibited (Fig. 3G). These data together indicated that deregulation of CX43 expression in the ovarian granulosa cells resulted in abnormal progesterone syntheses in female mice under cold exposure.

### PERK/NRF2 pathway activation was responsible for increased CX43 and StAR-dependent progesterone synthesis in the ovary of female mice under cold exposure

**Figure 4:**
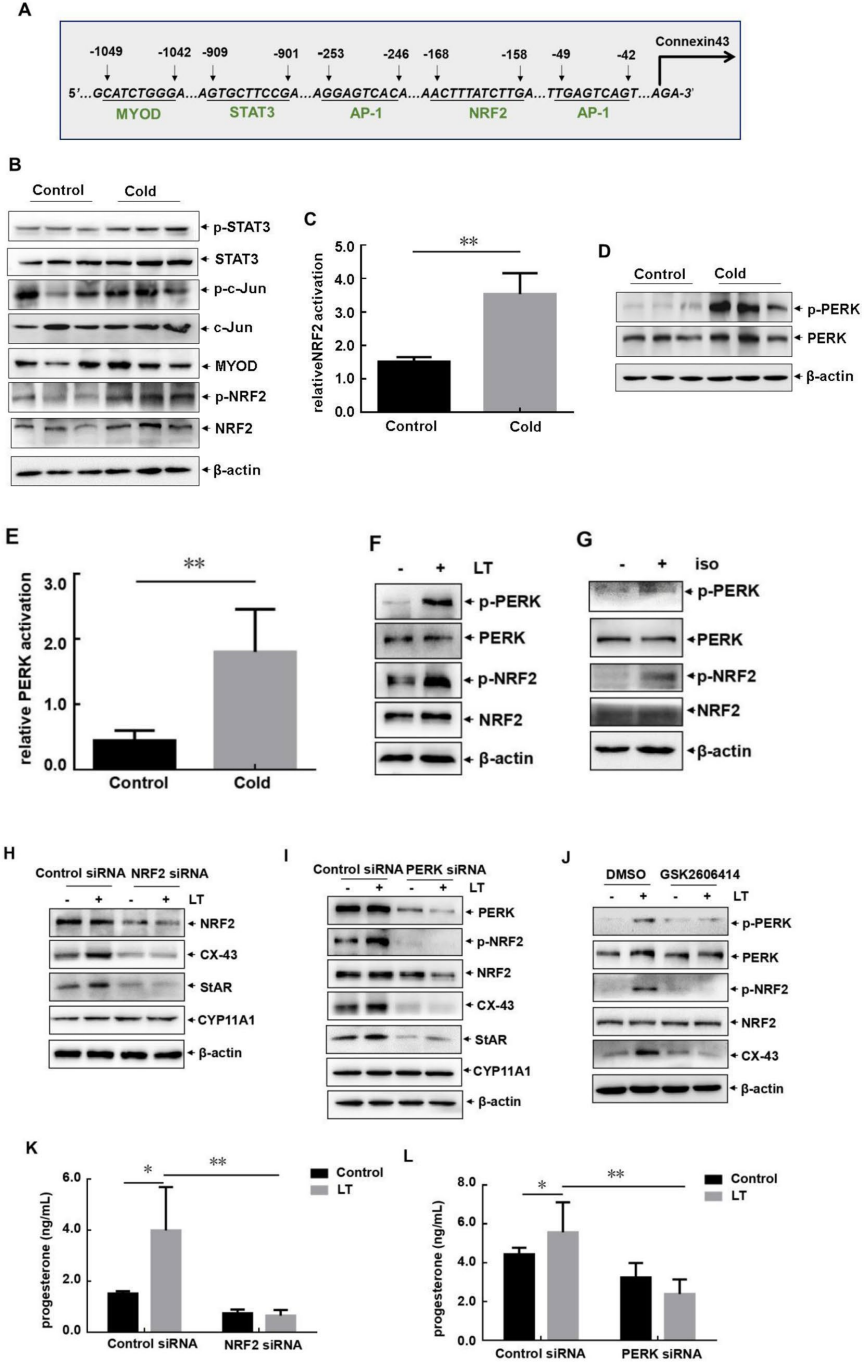
PERK/NRF2 pathway activation was responsible for increased CX43 and StAR-dependent progesterone synthesis in the ovary of female mice under cold exposure. (**A**) Potential cis-acting elements matching to several transcriptional factors (NRF2, STAT3, AP-1 and MyoD) were identified within mouse *CX43* promoter. (**B**) Female mice were untreated or exposed to cold stress as described in Fig. 1A. The ovaries of the mice in each group were collected and subjected to western-blot assay to detect the expression or activation levels of the above transcriptional factors. (**C**) The quantitative results of NRF2 activation (p-NRF2 relative to total NRF2) were shown (**p < 0.01). (**D**) The samples in B were used to detect the activation status of PERK. (**E**) The quantitative results of PERK activation (p-PERK relative to total PERK) were shown (**p < 0.01). (**F**,**G**) The primary granulosa cells (GCs) were extracted and treated as described in Fig. 2E,G. Then the activation status of PERK and NRF2 were detected. (H and K) The primary GCs were transfected with NRF2 siRNA or their control siRNAs, followed by exposure to cold stress for 12 h. Then the changes on CX43 and StAR expression (**H**) and the levels of progesterone in cell culture supernatants (**K**) were detected (**p < 0.01, *p < 0.05). (**I**,**L**) The primary GCs were transfected with PERK siRNA or their control siRNAs, followed by exposure to cold stress for 12 h. Then the changes on PERK/NRF2/CX43/StAR pathway activation (**I**) and the levels of progesterone in cell culture supernatants (**L**) were detected (**p < 0.01, *p < 0.05). (**J**) The primary GCs were pre-treated with PERK inhibitor GSK2606414 or its solvent DMSO, followed by exposure to cold stress for 12 h. Then the changes on PERK/NRF2/CX43/StAR pathway activation were detected.

To figure out the upstream signaling events leading to the enhancement of CX43 expression in the ovarian granulosa cells in response to cold exposure, we checked the cis-acting elements within mouse *CX43* promoter. Then DNA sequences matching to several transcriptional factors (NRF2, STAT3, AP-1 and MyoD) were identified (Fig. 4A). However, we only observed the increased phosphorylation of NRF2 in the ovary of cold-treated female mice (Fig. 4B,C); while activation/expression levels of c-Jun (major component of AP-1), STAT3 and MyoD did not show obvious changes under the same conditions (Fig. 4B). These data indicated the potential contribution of NRF2 to *CX43* transcription in the ovary.

Then we screened the upstream activator for NRF2, and a significant activation of the PERK, one of the endoplasmic reticulum (ER) stress sensor proteins, was observed in the ovary of female mice exposed to cold stress (Fig. 4D,E). In addition, both PERK and NRF2 activation were also observed in the granulosa cells treated by low temperature or exposed to isoproterenol (Fig. 4F,G). These data indicated that PERK and NRF2 might function as the upstream activators for mediating CX43 upregulation in the ovarian granulosa cells.

To address this possibility, NRF2 siRNA was transfected into the primary granulosa cells, and then we observed that blocking NRF2 expression efficiently inhibited the enhancement of CX43 transcription and protein synthesis induced by low temperature exposure (Fig. 4H), indicating that NRF2 was involved in transcriptional induction of *CX43* under cold condition. Furthermore, suppressing PERK expression (by its specific siRNA) or its activity (by its specific inhibitor GSK2606414) not only blocked the activation of NRF2, but also inhibited CX43 upregulation in response to low temperature treatment (Fig. 4I,J). Therefore, we concluded that PERK/NRF2 pathway activation was involved in upregulating CX43 expression in the ovary induced by cold.

We next focused on addressing whether PERK/NRF2 pathway activation contributed to regulating CX43-dependent downstream signaling events, enhancement of StAR-dependent progesterone synthesis in the ovary under cold exposure. Here we found that along with the suppression of CX43 upregulation in the PERK and NRF2 siRNA-transfected GCs, elevation of StAR expression was almost totally blocked; while of the levels of CYP11A1 did not change obviously under the same conditions (Fig. 4H,I). Moreover, enhancement of progesterone synthesis induced by low temperature treatment was also significantly inhibited in the granulosa cells under the same conditions (Fig. 4K,L). These data together indicated that cold exposure induced PERK/NRF2/CX43/StAR/progesterone pathway activation in the ovarian granulosa cells.

### Suppressing PERK expression in vivo blocked female reproductive disorders induced by cold exposure

**Figure 5:**
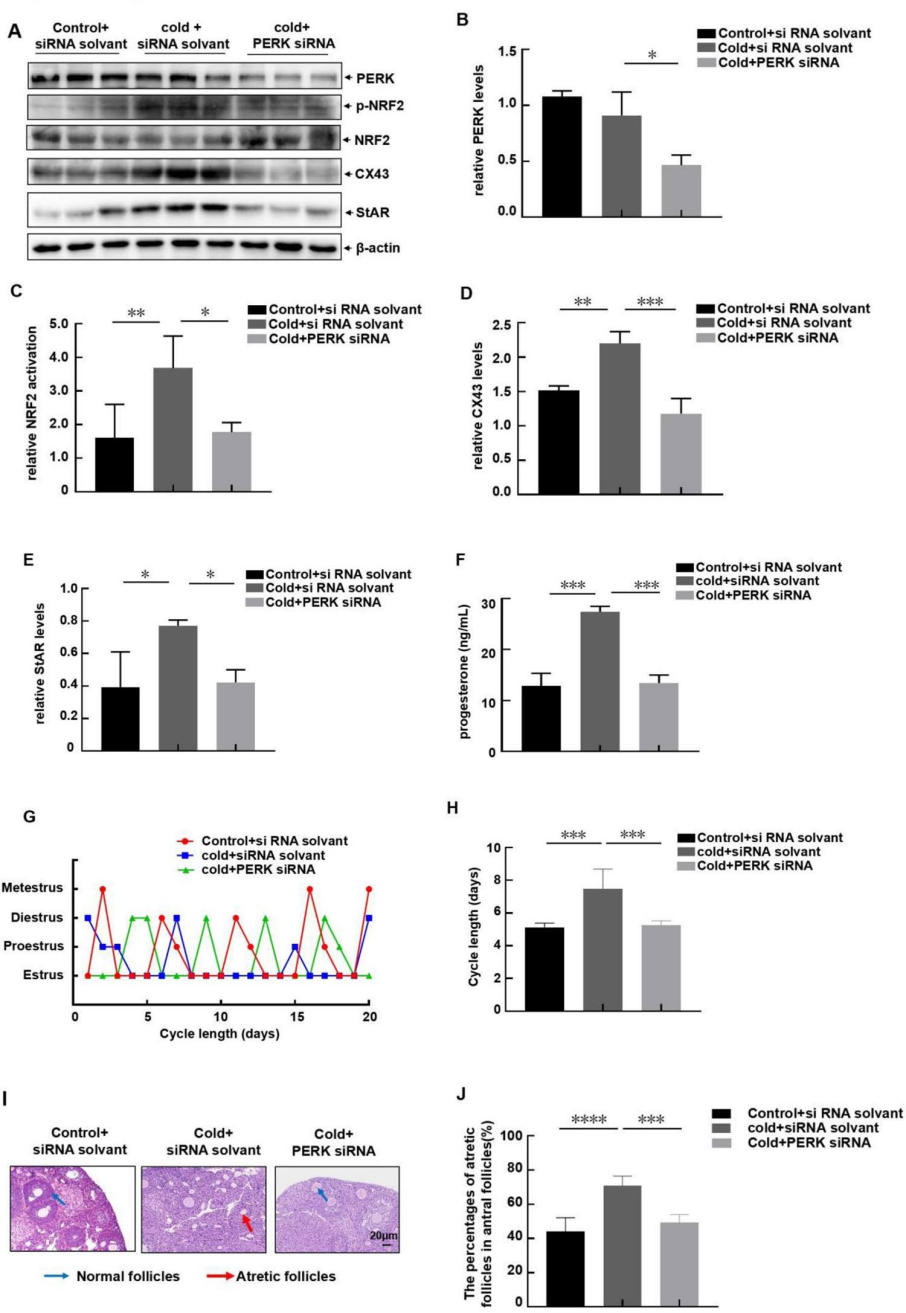
Suppressing PERK expression in vivo blocked female reproductive disorders induced by cold exposure. PERK or its control siRNA were given to the female mice by intraperitoneal injection (i.p) followed by exposing to cold stress. (**A**–**F**) The ovaries of the mice in each group were collected and subjected to western-blot assay (**A**) or ELISA (**F**) to detect the changes on PERK/NRF2/CX43/StAR/progesterone pathway activation. The quantitative results of PERK levels (relative to β-actin), NRF2 activation (p-NRF2 relative to total NRF2), CX43 and StAR levels (relative to β-actin) were shown in (**B**–**E**). The changes on serum levels of progesterone were shown in (**F**) (n = 6, *p < 0.05, **p < 0.01, ***p < 0.001). (**G**,**H**) The distribution of the estrous cycles of the mice in each group was detected in the following 3 weeks (**G**). And the average cycle length of mice in each group was shown in (**H**) (n = 10, ***p < 0.001). (**I**,**J**) The ovaries of the mice in each group were collected and subjected to HE staining. The follicles categorization was performed and the percentage of atretic follicles in the total follicles was calculated (n = 6, ***p < 0.001).

Finally, we tried to elucidate the contribution of ovarian PERK-dependent pathway activation to the reproductive disorders induced by cold exposure. To this end, PERK siRNA was given to the female mice by intraperitoneal injection (i.p) followed by exposing to low temperature. Then we found that in vivo administration of PERK siRNA significantly inhibited the expression level of ovarian PERK (Fig. 5A,B), accompanying with the suppression of NRF2 activation (Fig. 5A,C), CX43 and StAR upregulation (Fig. 5A,D,E), and the enhancement of progesterone synthesis (Fig. 5F) in the ovary after cold exposure. Under the same conditions, cold-induced estrous cycle prolongation was significantly inhibited, and the cycle length of PERK siRNA-treated mice recovered to the same level as that of the unstressed mice (Fig. 5G,H). In addition, administration of PERK siRNA also rescued the increase in follicular atresia induced by cold. The percentage of atresia follicles in mice treated with PERK siRNA decreased to almost the same level as the control mice (Fig. 5I,J). These data indicated that PERK/NRF2/CX43/StAR/progesterone pathway activation in the ovarian granulosa cells played a crucial role in mediating female reproductive disorders induced by cold exposure.

## Discussion

Cold environment is a health risk factor for female reproductive system. As more and more women work or travel in cold environments (such as high altitudes, polar region), the issue of how to maintain female reproductive health in low temperature cannot be ignored. In the current study, we focused on investigating the mechanisms involving in cold-induced female reproductive disorders. And our results indicated that aberrant activation of the PERK/NRF2/CX43/StAR/progesterone signaling pathway in the ovarian granulosa cells played a critical role in mediating estrous cycle prolongation and increased follicular dysplasia in the female mice upon cold exposure. Since most of the mechanistic studies focus on revealing the contribution of sympathetic activation and norepinephrine (NE) release to cold stress-induced responses in various organs (including ovarian disorders)^5,19^, our results have provided novel findings unrelated to the neural regulatory mechanism. Most importantly, we have also demonstrated that controlling the levels of ovarian PERK activation is validate for preventing reproductive disorders, thus providing potential strategy for maintaining female reproductive health under cold conditions.

In fact, PERK has been proved to be a cold-responsive signaling molecule in the previous reports^20–22^. Upon cold stimulation, the brown adipose tissue (BAT) that contains high abundance mitochondria can exhibit thermogenic capacity to maintain body temperature^19,23^. The active respiratory cristae formation inside the mitochondria of brown adipocytes is the key event for thermogenesis in response to low temperatures. As an ER-resident kinase, PERK is activated upon cold exposure and functions as a major regulator of mitochondrial cristae biogenesis and thermogenesis in BAT^21,23^. Therefore, activation of PERK in BAT is a key protective signaling event for enhancing cold adaptation. In contrast, cold stimulation-induced PERK activation in the foam cells leads to cell apoptosis and enhances the instability of atherosclerotic plaques, indicating that PERK functions as a stress mediator instead of a protector in the cardiovascular system^22^. Why PERK exhibits different functions in different tissue and cells in cold-induced responses? We notice that PERK is involved in thermogenesis in BAT by activating ER stress-unrelated and eIF2α/ATF4-independent pathway^20^; while the pro-apoptotic role of PERK in foam cells is mediated by promoting the activation of eIF2α/ATF4/CHOP signaling cascade^22^. Similarly, PERK/eIF2α/ATF4/CHOP signaling pathway activation has also been proved to play a pivotal role in GCs apoptosis associated with ER stress activation in the ovary under both physiological and stress conditions^13–17^. In the current study, we have revealed that another well-known PERK substance NRF2 was activated in response to cold stress. Therefore, our results threw novel insights into the mechanism of PERK involving in regulating cold stress-induced health risk.

Oxidative stress is a typical response under cold exposure. As a master anti-oxidant regulator, NRF2 is activated in various tissues and cells provoked by cold stress^24–28^. Typically, activated NRF2 can exert a protective role to remove injurious oxidative stress and improve pathological conditions^24^. For example, upregulation of NRF2 expression is regarded as a critical signaling event to cope with cold-induced liver injury^25^. In addition, increased NRF2 acetylation in the colon induces an anti-oxidant response to maintain intestinal homeostasis following cold exposure^26^. In the central nervous system, cold stimulation induces NRF2-dependent anti-oxidative genes expression, which can provide the protective effect on hippocampal neuronal apoptosis^27^. In addition to its anti-oxidative and cytoprotective roles, NRF2 is also involved in regulating thermogenesis and energy expenditure upon cold stress conditions. However, NRF2 activation induced by low temperature blocks the browning of white adipose tissue and adipogenesis^28^. These data indicate that activating NRF2 under cold stress is a harmful response to energy metabolism. In the current study, we identified the new function of NRF2 in regulating the ovarian disorders followed by cold exposure. These data are new evidence for the function of NRF2 in mediating cold-induced health risk response in the female reproductive system.

The transcription target of NRF2 in mediating cold-induced ovarian dysfunction is CX43, which is necessary to form gap junctions and regulate the nutrient transport in follicles and maintain the oocyte in a state of meiotic arrest under physiological conditions. Regularly, CX43 levels transiently increased by LH surge before ovulation and then decreased after ovulation, which event is critical for attenuating the communication between the oocyte and granulosa cells and allowing the oocyte to complete meiosis^9^. However, CX43 levels in the ovarian granulosa cells increased significantly following cold exposure. That is obviously an inhibitory signal for the maturation of oocytes. For another point, CX43 is proved to mediate cold-induced white adipose tissue browning by facilitating the propagation of sympathetic neuronal signals^29^. Therefore, enhanced intercellular communication mediated by CX43 may also occur in ovarian granulosa cells and augment the ovarian dysfunctions induced by cold stress.

The development of ovarian follicles is a hormone dependent process. Progesterone has the property to inhibit follicle growth by slowing the rate of mitosis^22,24,25^. In a previous report, strong evidence supported that cold stress causes an increase in plasma progesterone levels, accompanying by ovarian morphological changes related to PCOS. However, no changes in plasma estradiol levels were detected under the same cold stress conditions^2^. These data are consistent with the results obtained in our current study. Notably, data in the previous study demonstrated that increase in progesterone expression induced by cold stress is mediated by the neuropeptide RFRP-3 expressed in both the hypothalamus and ovary, which acting on components of the sterogenesis pathway downstream from StAR^2^. Our results disclosed the novel CX43 upregulation-dependent signaling pathway leading to increased expression of StAR in granulosa cells, thereby adding new evidence regarding the mechanism leading to hormone disorders provoked by cold stress.

## Conclusion

**Figure 6:**
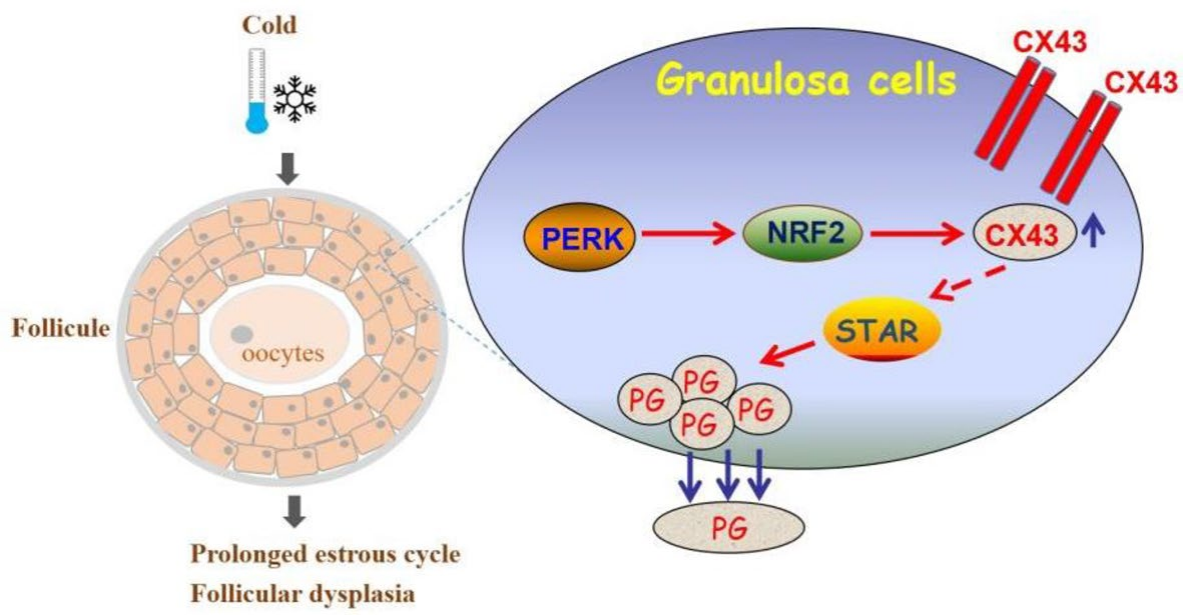
Summary paradigm. Cold exposure induced PERK/NRF2/CX43/StAR/progesterone pathway activation in the ovarian granulosa cells, which played a crucial role in mediating estrous cycle prolongation and follicular dysplasia in female mice.

In sum, we have disclosed a previously unidentified signaling pathway (PERK/NRF2/CX43/StAR/progesterone) involving in regulating female reproductive disorders in response to cold stress (Fig. 6). Moreover, we have also found potential strategy for maintaining female reproductive health under cold conditions by targeting the ovarian protein kinase PERK, thus providing new insights into improving women’s health in extreme environments.

### Supplementary Information

Supplementary Figures.

## Data Availability

The datasets used and/or analyzed during the current study are available from the corresponding author on reasonable request.
