# Supplementary figures and images for: Ovarian PERK/NRF2/CX43/StAR/progesterone pathway activation mediates female reproductive dysfunction induced by cold exposure
Source: Sci Rep. 2024 May 4;14:10248. doi: 10.1038/s41598-024-60907-9 (PMC11068861; doi:10.1038/s41598-024-60907-9)

**Figure 2**

**2A**

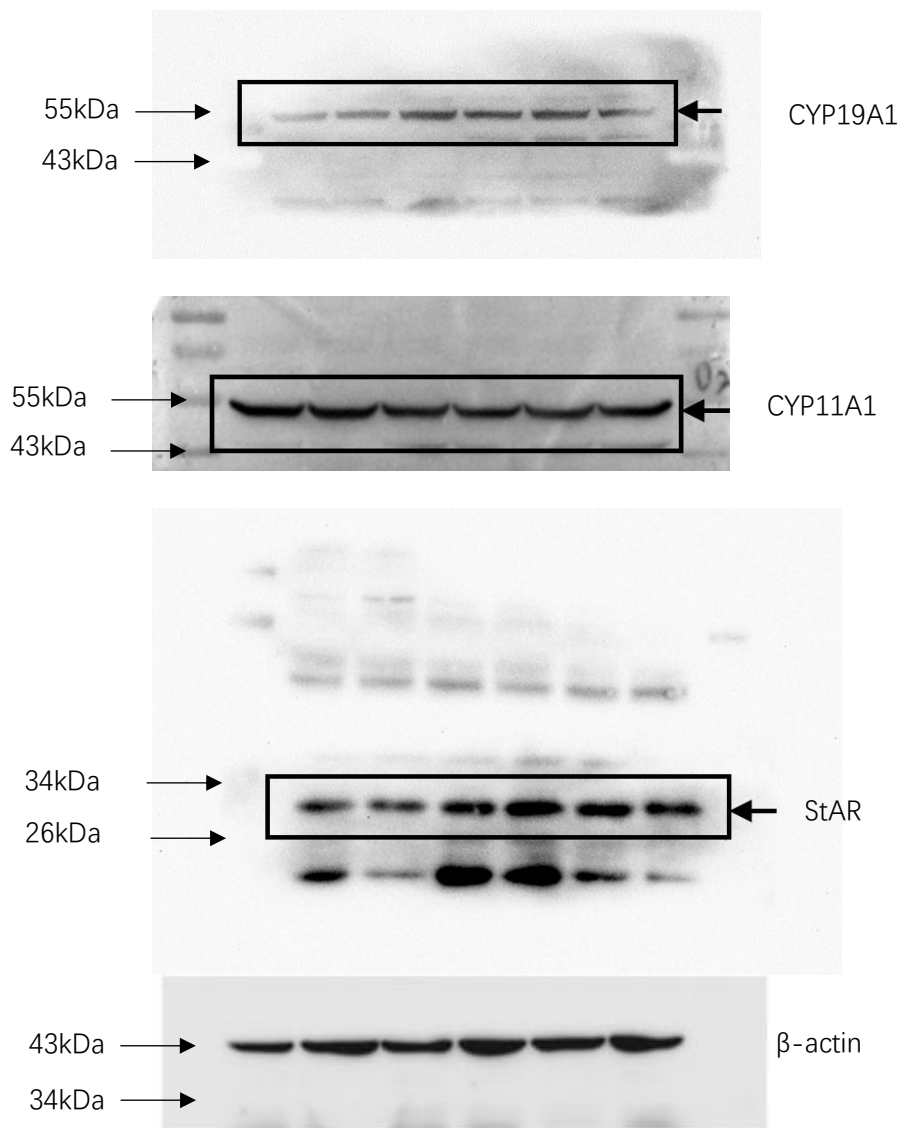

2C

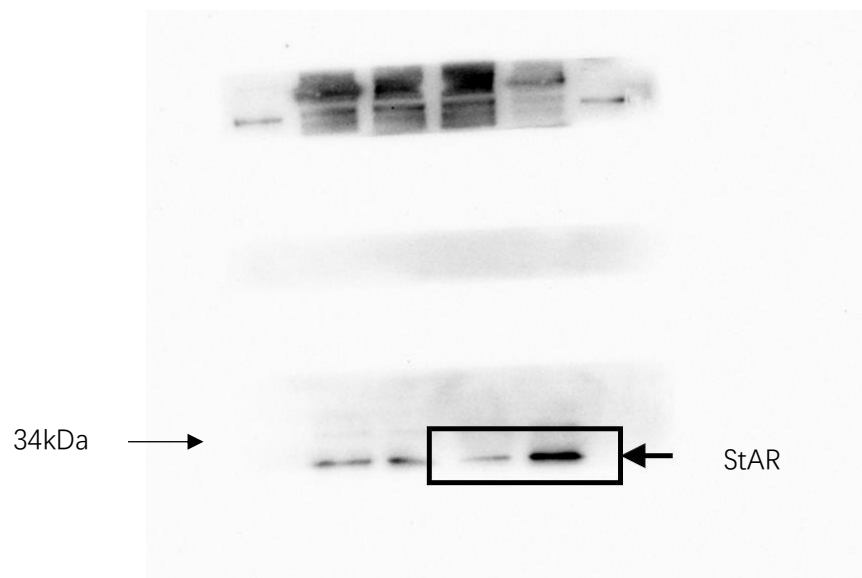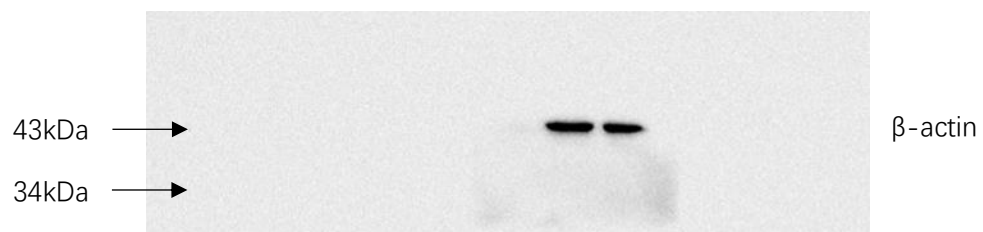

2G

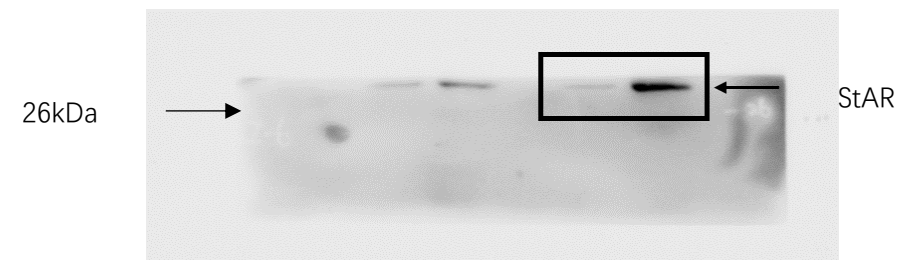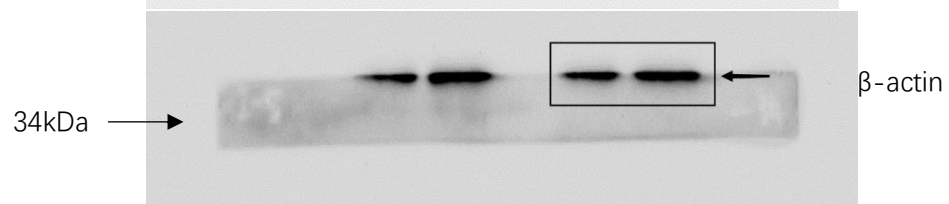

**Figure 3**

**3A**

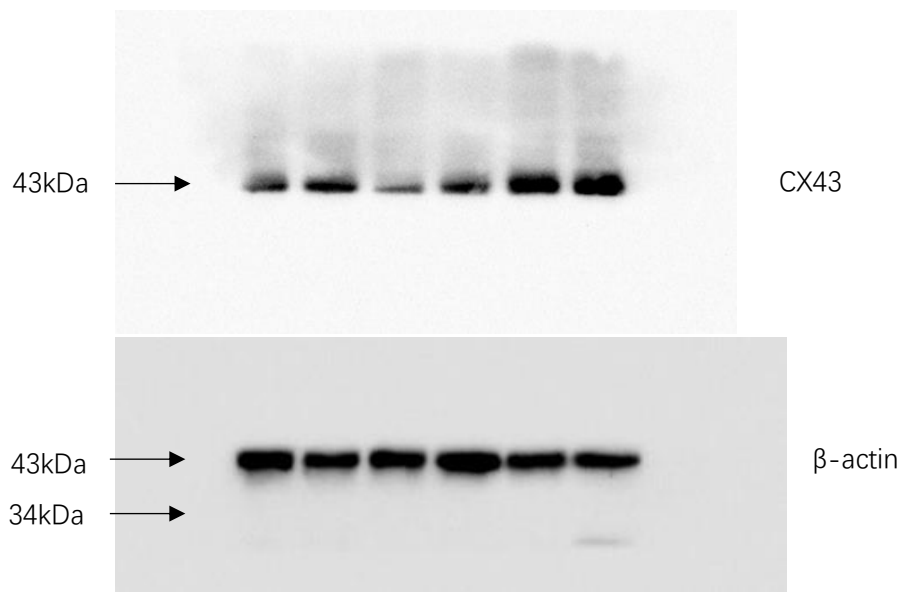

**3D**

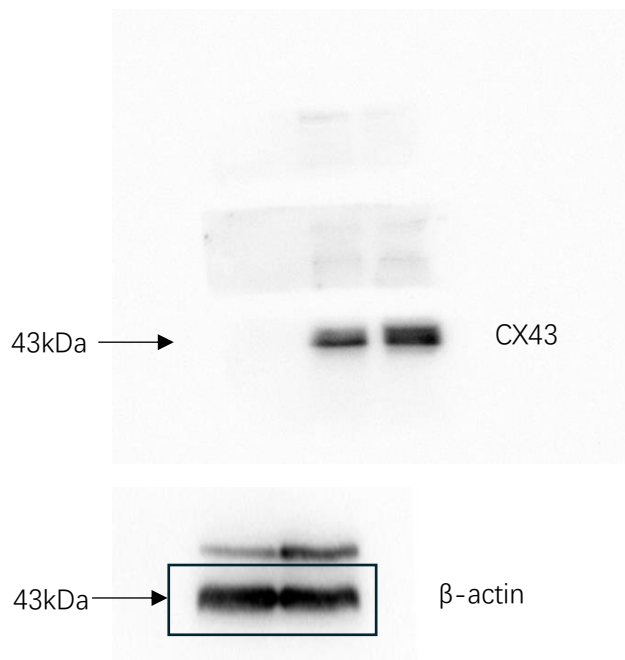

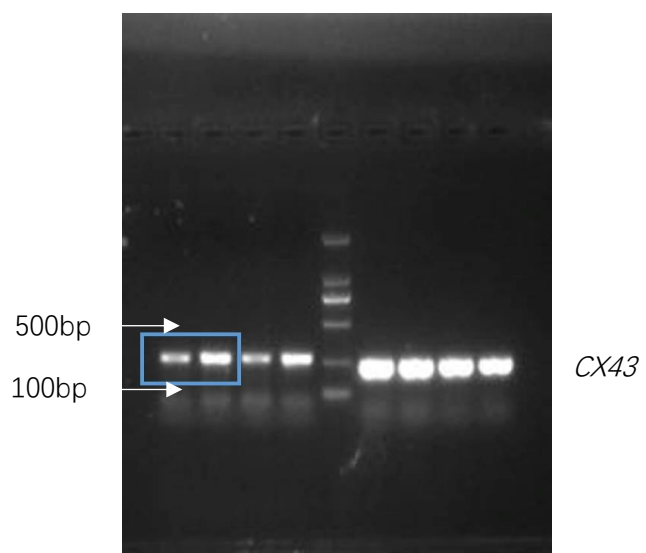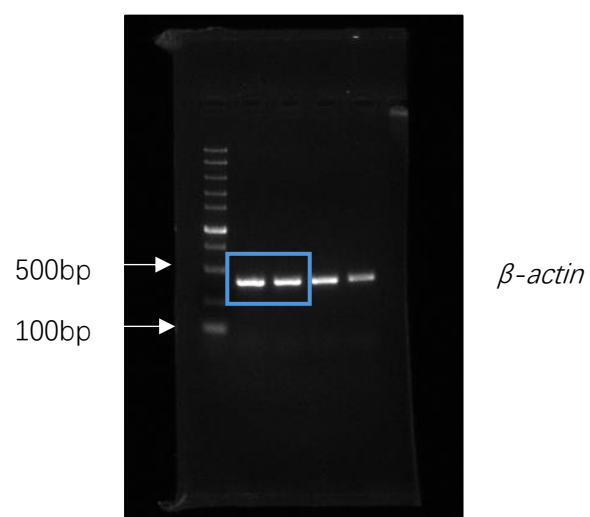

3E

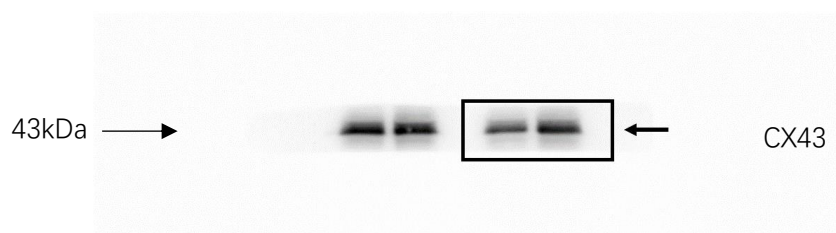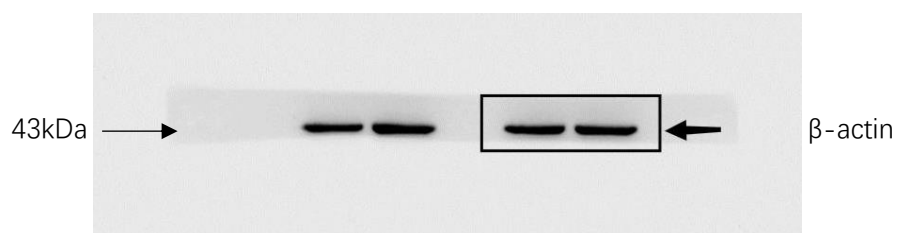

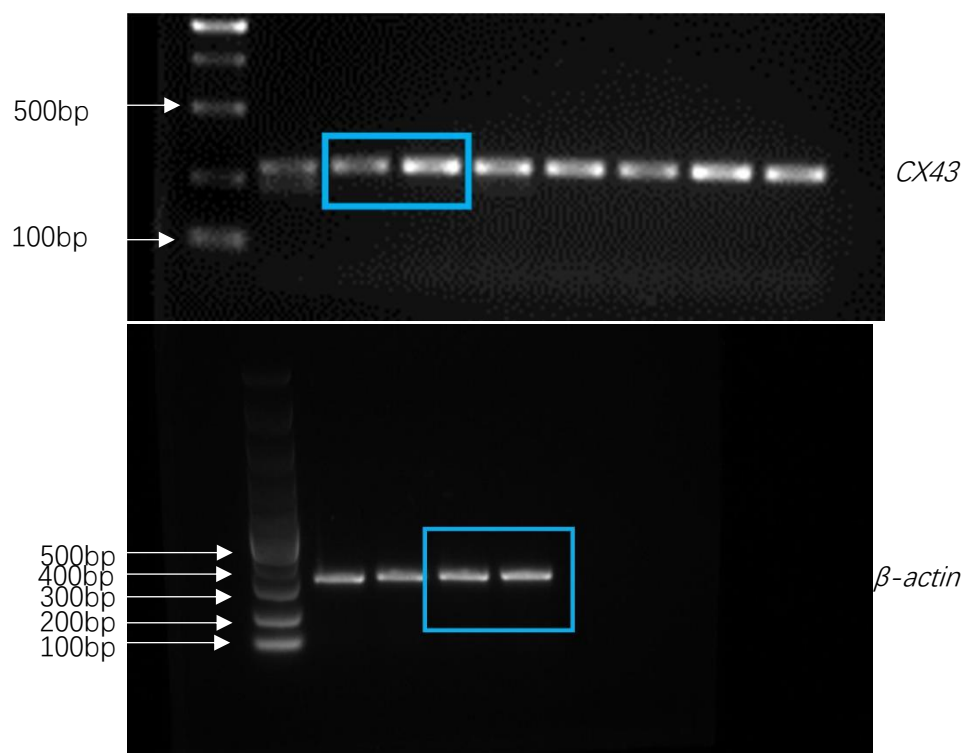

3F

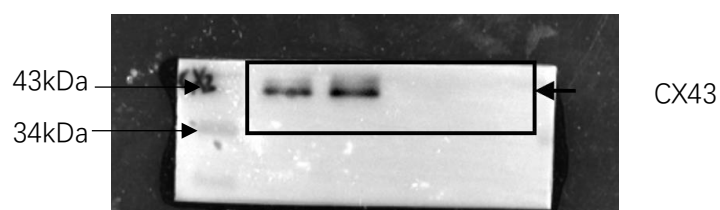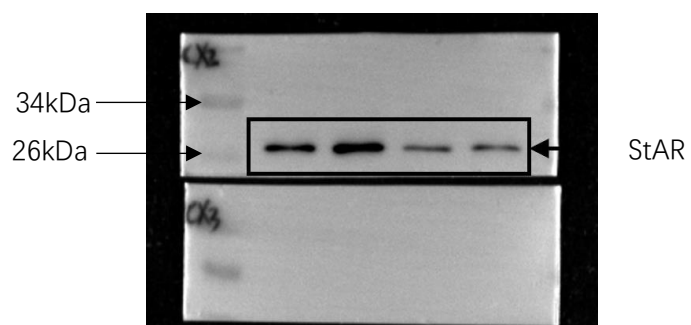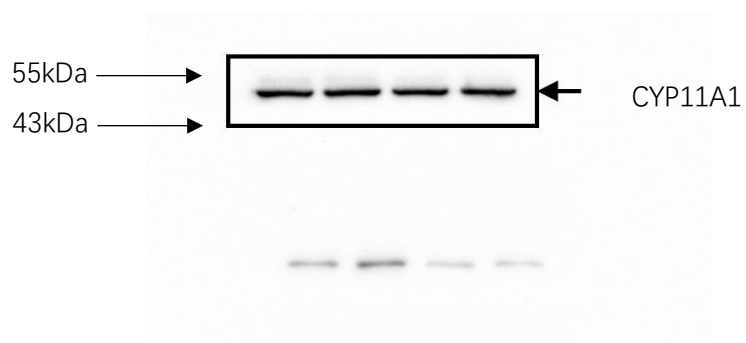

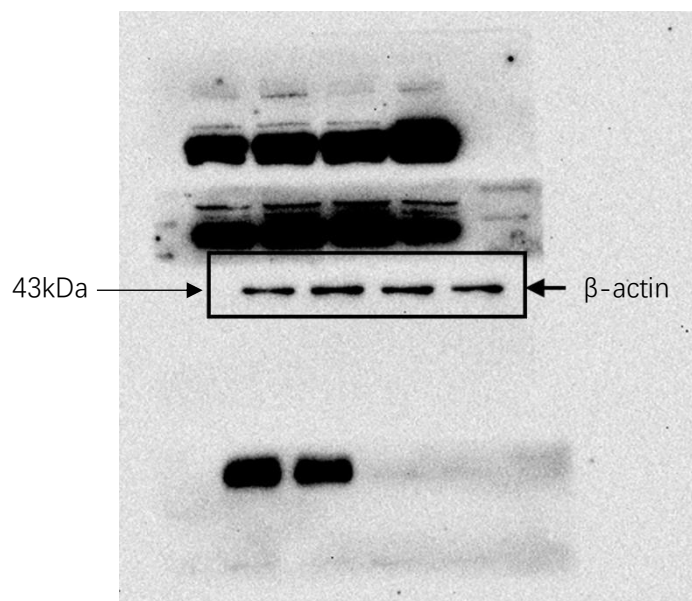

**Figure 4**

**4B**

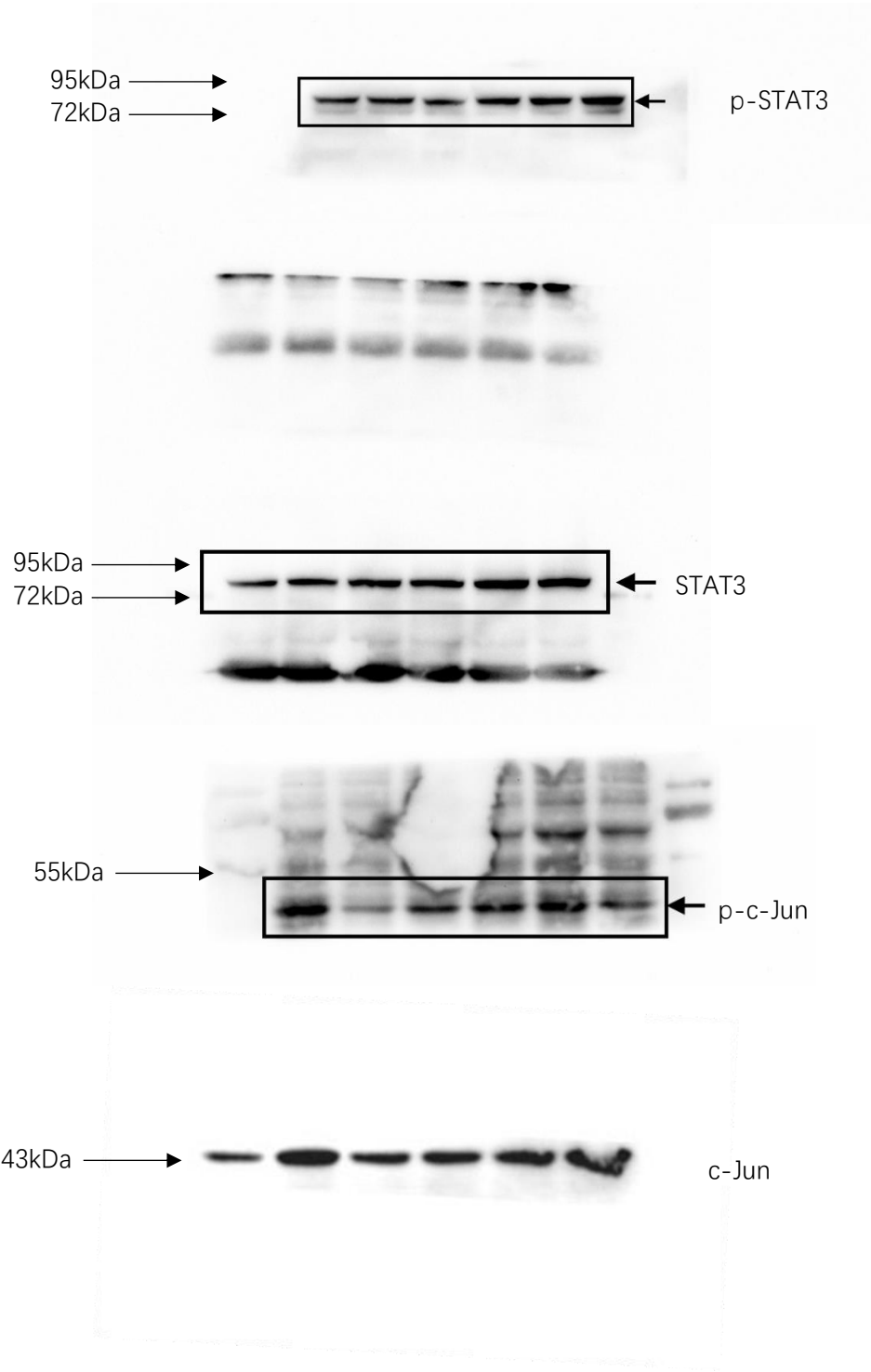

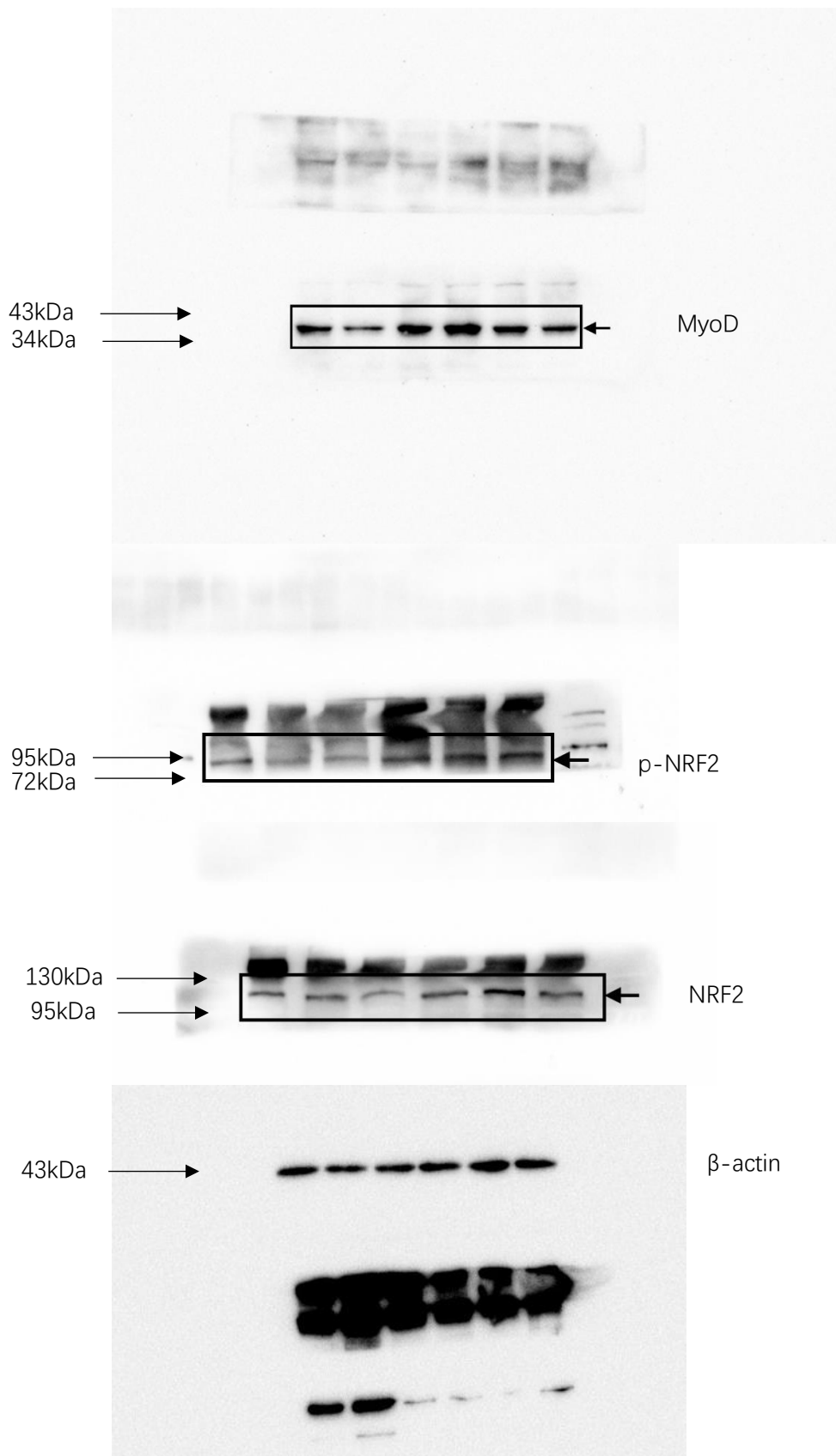

**4D**

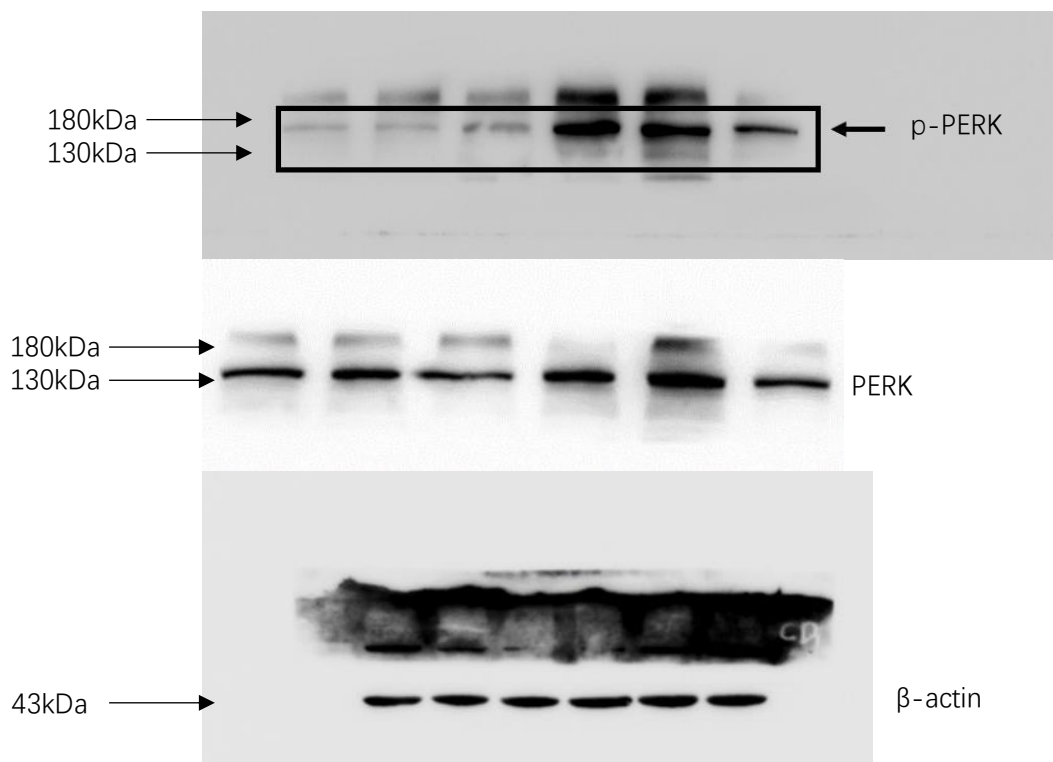

**4F**

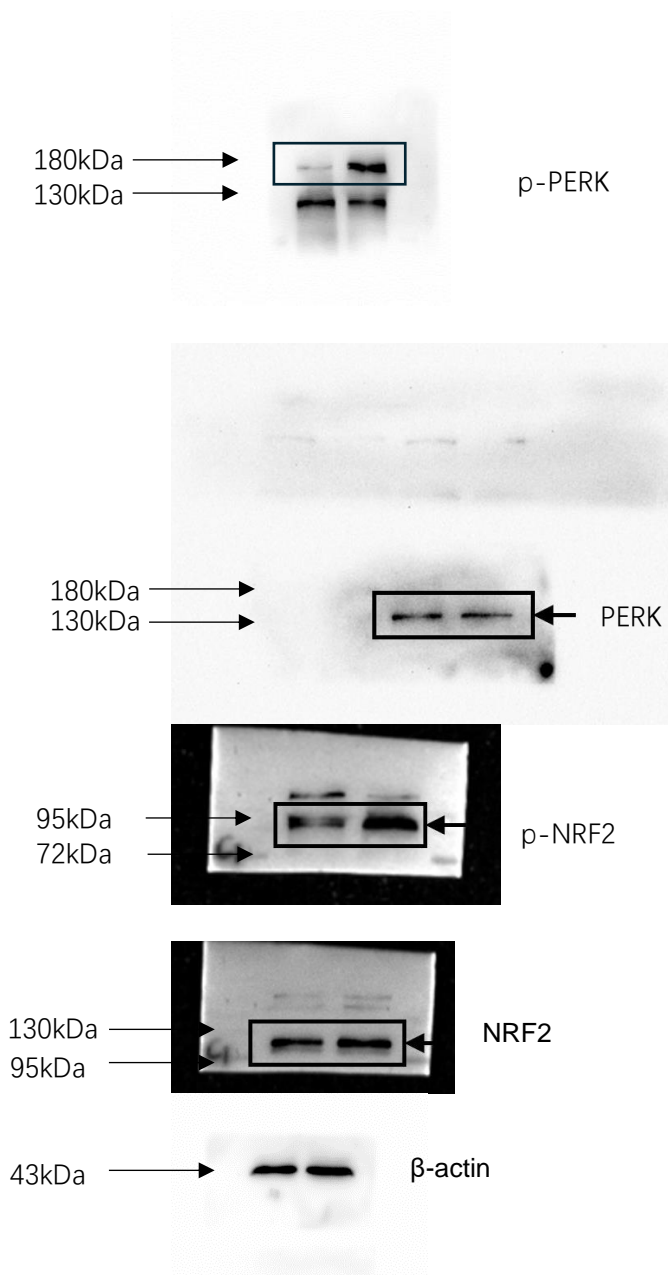

**4G**

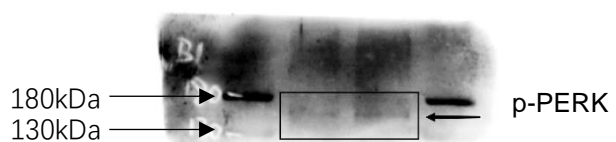

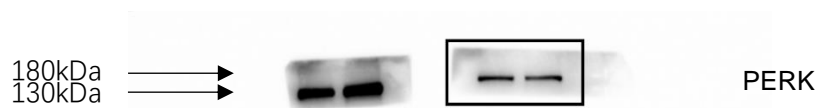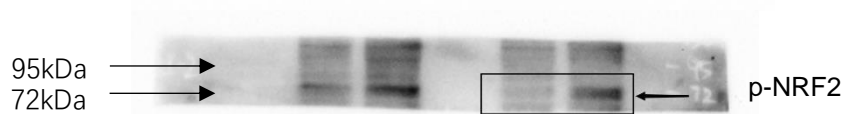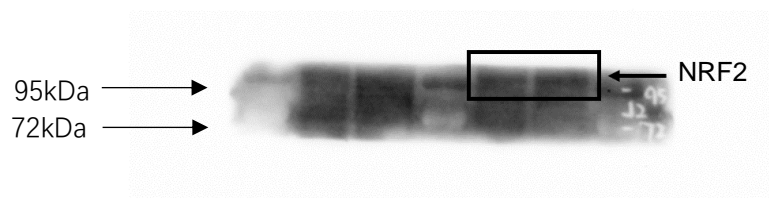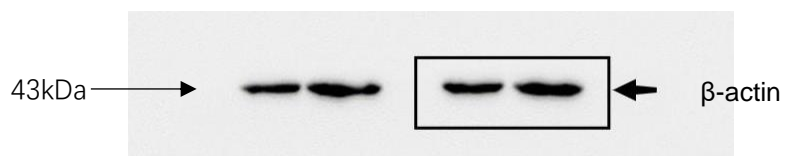

**4H**

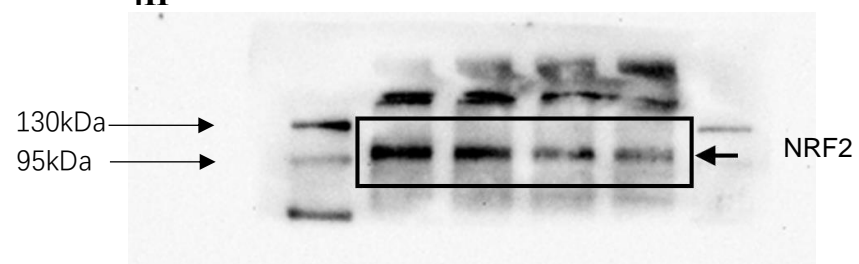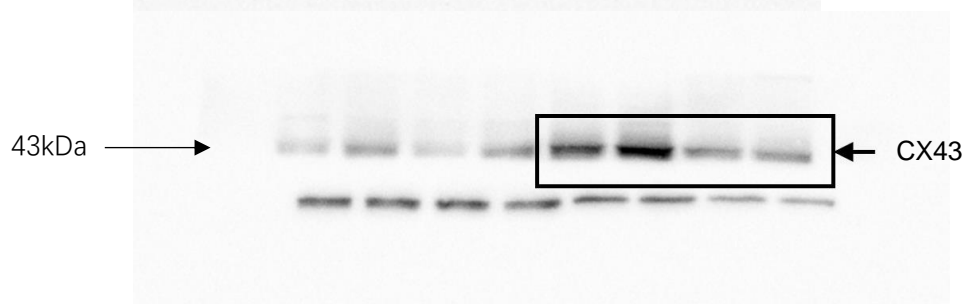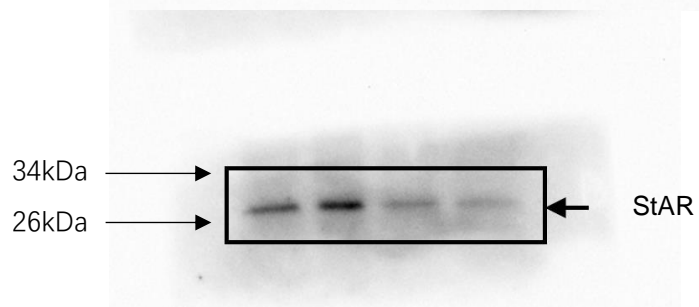

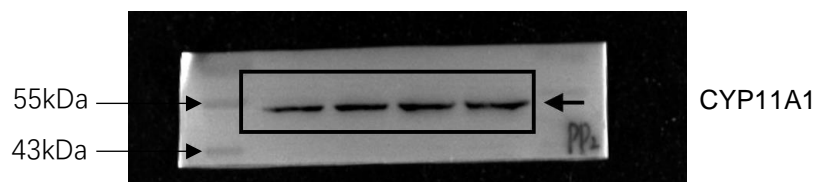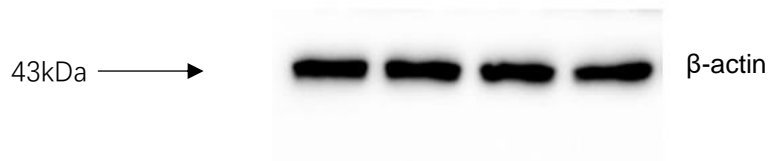

**4I**

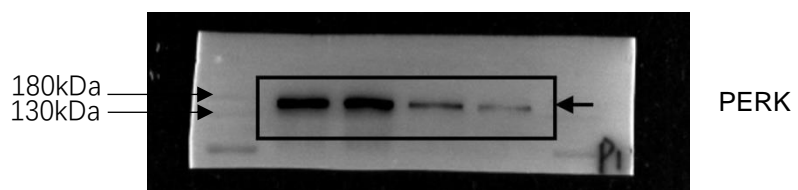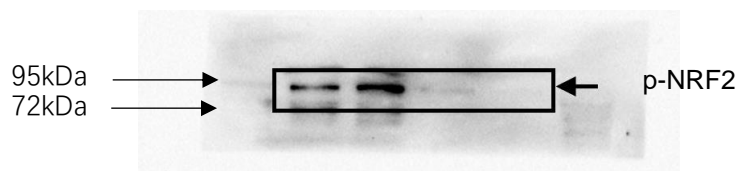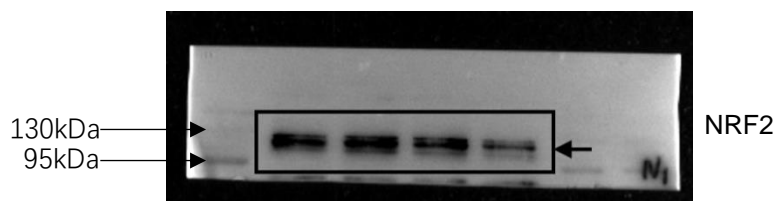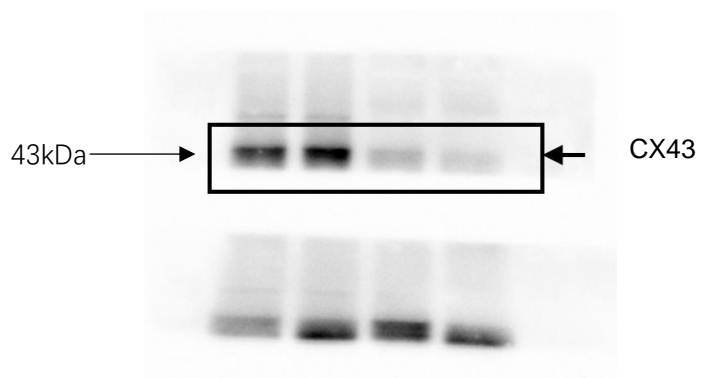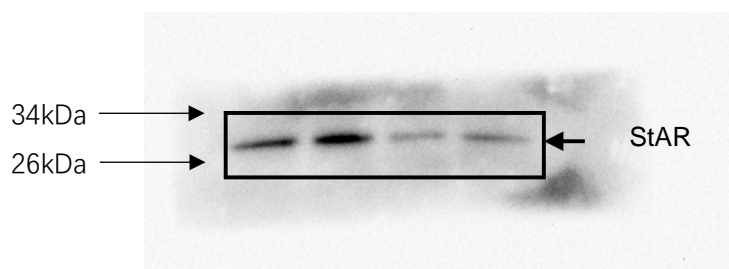

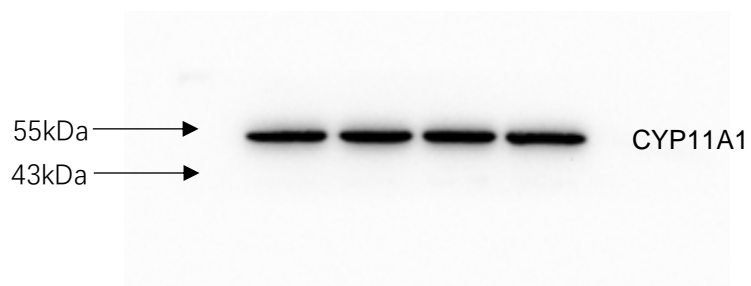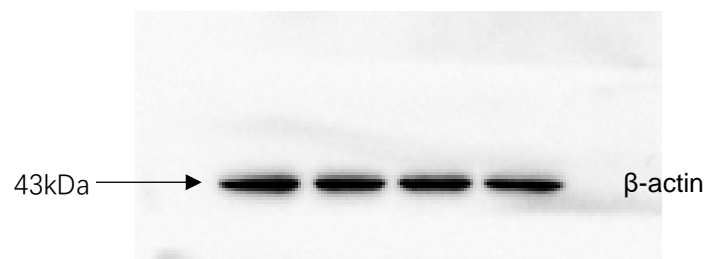

**4J**

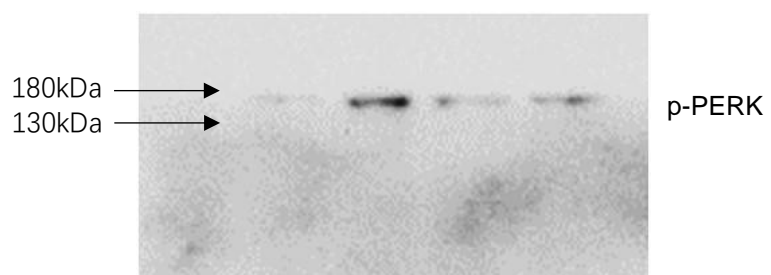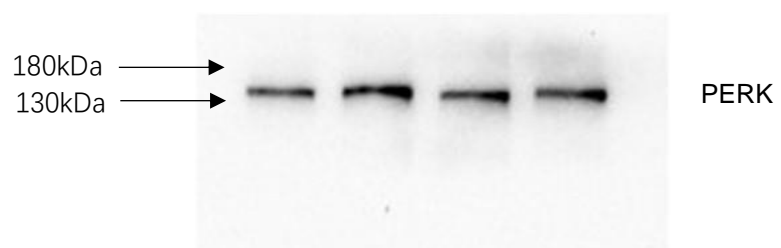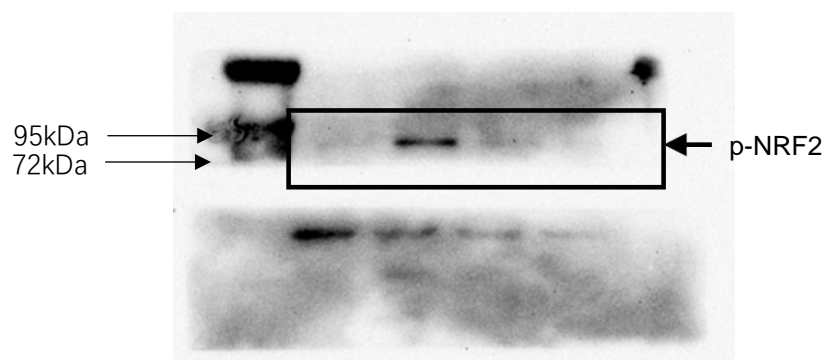

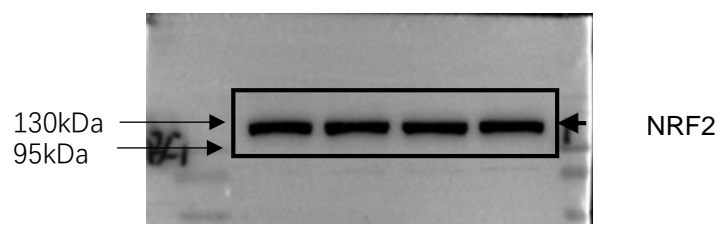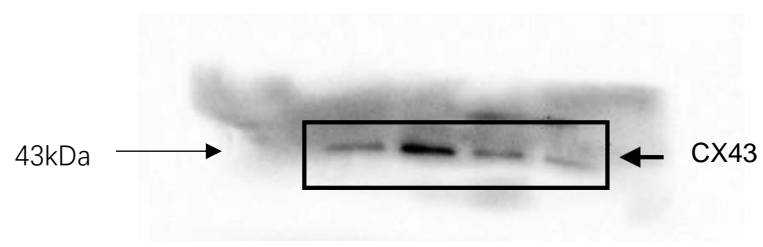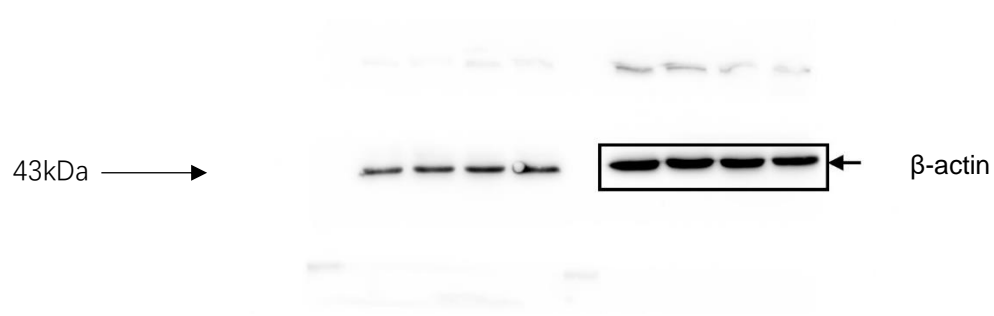

Figure 5

5A

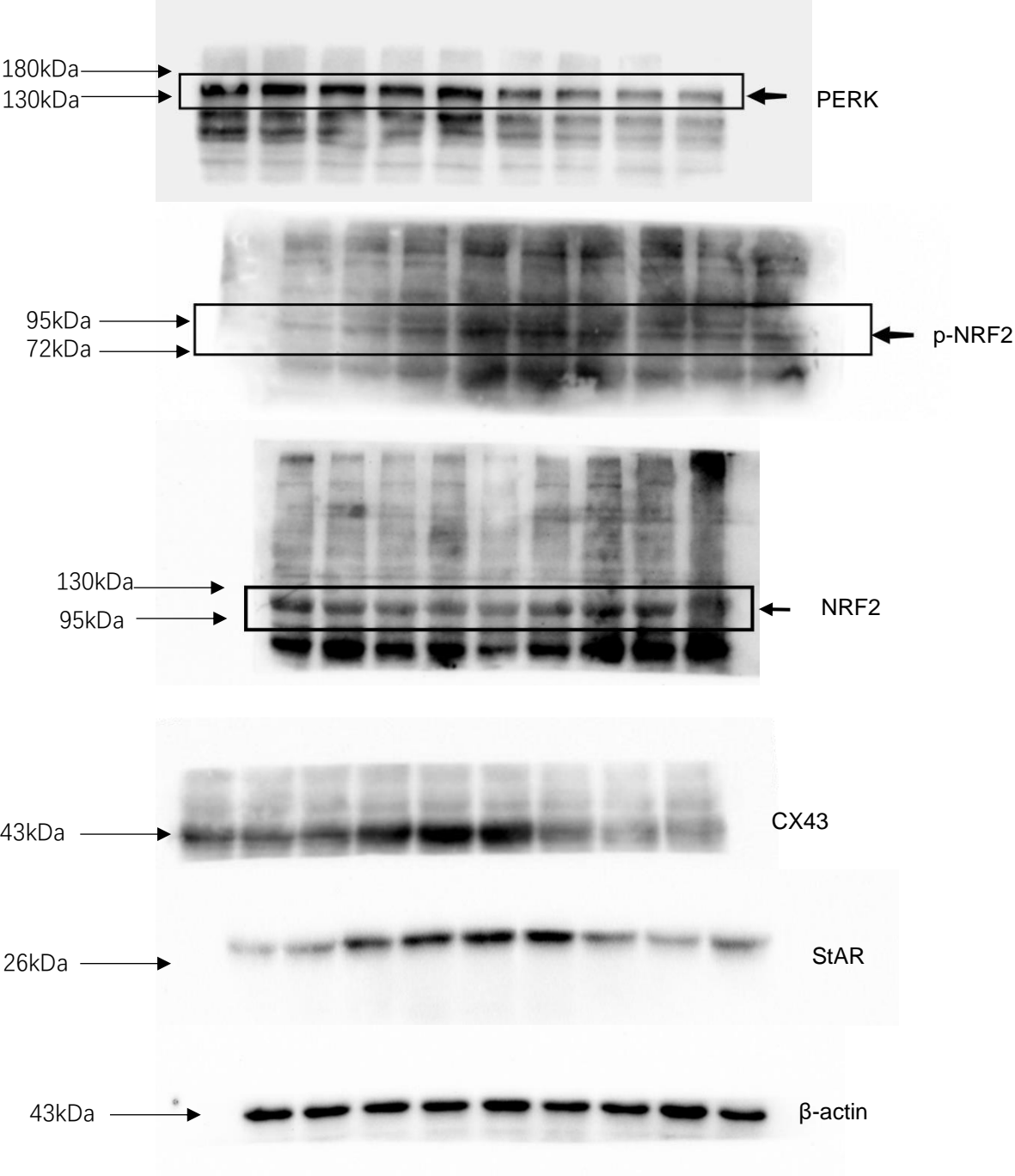

Supplement: Supplementary file 1 — Supplementary Figures. [file 41598_2024_60907_MOESM1_ESM.pdf]
